# EANM practice guideline for quantitative SPECT-CT

**DOI:** 10.1007/s00259-022-06028-9

**Published:** 2022-12-05

**Authors:** John C. Dickson, Ian S. Armstrong, Pablo Minguez Gabiña, Ana M. Denis-Bacelar, Aron K. Krizsan, Jonathan M. Gear, Tim Van den Wyngaert, Lioe-Fee de Geus-Oei, Ken Herrmann

**Affiliations:** 1 Institute of Nuclear Medicine, University College London Hospitals Foundation Trust, London, UK; 2 Nuclear Medicine, Manchester University NHS Foundation Trust, Manchester, UK; 3 Department of Medical Physics and Radiation Protection, Gurutzeta-Cruces University Hospital/Biocruces Health Research Institute, Barakaldo, Spain; 4 Department of Applied Physics, Faculty of Engineering, UPV/EHU, Bilbao, Spain; 5 National Physical Laboratory, Teddington, UK; 6 ScanoMed Nuclear Medicine Centers, Debrecen, Hungary; 7 Joint Department of Physics Institute of Cancer Research and Royal Marsden, NHS Foundation Trust, Sutton, Surrey UK; 8 Department of Nuclear Medicine, Antwerp University Hospital, Edegem, Belgium; 9 Faculty of Medicine and Health Sciences (MICA – IPPON), , University of Antwerp, Wilrijk, Belgium; 10 Department of Radiology, Section of Nuclear Medicine, Leiden University Medical Center, Leiden, The Netherlands; 11 Biomedical Photonic Imaging Group, University of Twente, Enschede, The Netherlands; 12 Department of Nuclear Medicine, University of Duisburg-Essen, and German Cancer Consortium (DKTK)-University Hospital Essen, Essen, Germany

**Keywords:** SPECT-CT, Quantification, Dosimetry, Bone, Neurology, Cardiology

## Abstract

**Purpose:**

Quantitative SPECT-CT is a modality of growing importance with initial developments in post radionuclide therapy dosimetry, and more recent expansion into bone, cardiac and brain imaging together with the concept of theranostics more generally. The aim of this document is to provide guidelines for nuclear medicine departments setting up and developing their quantitative SPECT-CT service with guidance on protocols, harmonisation and clinical use cases.

**Methods:**

These practice guidelines were written by members of the European Association of Nuclear Medicine Physics, Dosimetry, Oncology and Bone committees representing the current major stakeholders in Quantitative SPECT-CT. The guidelines have also been reviewed and approved by all EANM committees and have been endorsed by the European Association of Nuclear Medicine.

**Conclusion:**

The present practice guidelines will help practitioners, scientists and researchers perform high-quality quantitative SPECT-CT and will provide a framework for the continuing development of quantitative SPECT-CT as an established modality.

## Preamble

The European Association of Nuclear Medicine (EANM) is a professional non-profit medical association that facilitates communication worldwide among individuals pursuing clinical and research excellence in nuclear medicine. The EANM was founded in 1985. These guidelines are intended to assist practitioners in providing appropriate nuclear medicine care for patients. They are not inflexible rules or requirements of practice and are not intended, nor should they be used, to establish a legal standard of care. The ultimate judgment regarding the propriety of any specific procedure or course of action must be made by medical professionals taking into account the unique circumstances of each case. Thus, there is no implication that an approach differing from the guidelines, standing alone, is below the standard of care. On the contrary, a conscientious practitioner may responsibly adopt a course of action different from that set out in the guidelines when, in the reasonable judgment of the practitioner, such course of action is indicated by the condition of the patient, limitations of available resources or advances in knowledge or technology subsequent to publication of the guidelines. The practice of medicine involves not only the science but also the art of dealing with the prevention, diagnosis, alleviation and treatment of disease.

The variety and complexity of human conditions make it impossible to always reach the most appropriate diagnosis or to predict with certainty a particular response to treatment. Therefore, it should be recognised that adherence to these guidelines will not ensure an accurate diagnosis or a successful outcome. All that should be expected is that the practitioner will follow a reasonable course of action based on current knowledge, available resources and the needs of the patient to deliver effective and safe medical care. The sole purpose of these guidelines is to assist practitioners in achieving this objective.

## Introduction

In nuclear medicine, gamma cameras and SPECT-CT systems are routinely used for quantitative imaging. From determining relative kidney performance, to binding ratios in the brain, one of the strengths of gamma camera imaging is its ability to quantify in-vivo physiology for a wide range of conditions and applications. While traditional gamma camera imaging has focused mostly on relative uptake of the radiopharmaceutical, for example to the opposing kidney, or specific structures in the brain, PET imaging has focused on absolute quantification in organs or features, producing images with quantitative units such as kBq/mL or standardised uptake value (SUV). This has brought advantages. The ability to perform absolute quantification, with ^18^F-FDG for example, provides the opportunity to assess the metabolic status of the disease for diagnosis, staging, treatment monitoring and disease progression.

For many years, SPECT-CT imaging has been perceived to be the poor relative of PET-CT for quantitative imaging. However, equipment and software developments including the incorporation of measured CT-based attenuation correction, scatter correction and correction for partial volume effects, have made significant progress in improving SPECT-CT quantification [1]. Using modern techniques, quantification of SPECT-CT data is now possible in a similar way as it is in PET-CT. However, quantitative SPECT-CT offers many advantages over PET-CT, which has the potential to lead to a wider range of applications. SPECT uses longer physical half-life radiopharmaceuticals that can better match biological processes. While longer half-life radionuclides such as ^64^Cu, or ^124^I are available in PET, these have inferior imaging characteristics in terms of their positron emission probability and high effective dose [2, 3], which diminish some of the advantages of PET imaging. A further advantage of SPECT is that its radiopharmaceuticals can be labelled with different radionuclides, offering the possibility to image multiple physiological processes at the same time. Studies with CZT SPECT systems are showing the ability to perform simultaneous ^99m^Tc/^123^I imaging [4], which, for example, could be applied in simultaneous perfusion/innervation studies in the heart, and perfusion/DaT (dopamine transporter) availability in the brain. But it is the widespread availability of SPECT-CT and the wide range of accompanying radiopharmaceuticals which provides the greatest advantage of quantitative SPECT-CT over PET-CT, with much wider global market penetration due to the lower cost of imaging equipment, and the straightforward radiopharmaceutical production.

Although at an early stage, the applications of quantitative SPECT-CT are becoming clearer. The driving force in the increasing interest in quantitative SPECT-CT comes from the growth in radionuclide therapy and theranostics, and the associated growth in personalised treatment planning. The unique advantage of many nuclear medicine therapies employing gamma-emitting therapeutic radionuclides is that they allow us to image and quantify the radiopharmaceutical bio-distribution after administration by SPECT-CT imaging. Because of this, internal absorbed dose calculations of radionuclide therapies using current commonly used therapeutic radionuclides such as ^177^Lu and ^131^I [5, 6] present the opportunity to verify treatment delivery and/or personalise the treatment over the course of multi-cycle administrations. Furthermore, imaging surrogates used with quantitative SPECT-CT can be used for diagnosis and patient selection prior to radionuclide therapy, in addition to assessment of disease progression [7, 8] or treatment response. Applications in the quantification of bone tracers for orthopaedic [9, 10] and cancer applications [11] are developing, while opportunities in cardiac [12] and neurological [13] imaging also exist.

Given these early, but rapidly developing applications, it is vital that integration of such technology into clinical practice is performed correctly. This is necessary in terms of correct image interpretation and to facilitate the pooling of data to improve scientific knowledge. Furthermore, for successful adoption, it is necessary that techniques are robust, consistent and correctly understood by the user for their application. There must also be clear applications of the technology. For example, quantitative SPECT-CT may enable a better understanding of why a certain subgroup of patients does not benefit from radionuclide therapy despite adequately expressing the target. And in the development of new radionuclide therapies with a narrower therapeutic index, quantitative SPECT-CT has the potential to support clinical development in identifying the optimal activity to be administered to each patient. The incremental value of using quantitative SPECT-CT for clinical care needs, however, still needs to be demonstrated. A definition of how to appropriately perform quantitative SPECT-CT is therefore essential to provide a stable platform for the understanding and application of this technology.

These guidelines have been written with the objective of defining standards for quantitative SPECT-CT. Reviewing the current status of quantitative imaging in SPECT-CT and highlighting limitations and areas in need of development, we demonstrate where quantitative imaging is potentially most beneficial. In these guidelines, we describe the procedural considerations that must be met in both achieving and implementing quantitative measurements for SPECT imaging and provide clinical applications for which its implementation has already been successful.

## Imaging protocols for quantitative SPECT-CT

### Overview

The scope of this section is to provide technical recommendations on image acquisition and reconstruction to achieve reliable quantitative SPECT-CT images. They are intended to be used with gamma cameras with conventional planar detectors rotating around the patient during the SPECT acquisition. Other gamma camera designs such as those with solid state detectors or novel non-parallel collimation are not covered within these guidelines, although some of the concepts described are still relevant.

The primary aim for quantitative SPECT-CT is to produce a tomographic image with voxel values representing activity concentration. The utilisation of these voxel values will then be dependent on the clinical application. For radionuclide dosimetry (for diagnostic and therapeutic applications), the absolute activity in Becquerels (Bq) within a delineated volume or organ is extracted from the images, and hence accuracy is paramount. For other diagnostic applications, voxel values may be converted to standardised uptake values (SUV). In this latter example, the SUV should translate to a clinically relevant biomarker and arguably the reproducibility of this metric is more relevant than its absolute accuracy. This is particularly pertinent to follow-up studies monitoring disease response to treatments.

While activity concentration for dosimetry is almost always defined in a volume representing an organ or cancerous deposit, SUV can be measured using several metrics. Like in dosimetry, the mean value of SUV (SUV_mean_) in a feature can be defined. However, SUV_max_ is more commonly used as a better representation of the intensity of uptake in a feature, even with its limitation of being reliant on a single pixel value and therefore being more susceptible to image noise affecting both the bias and precision of the measure. SUV_peak_ is an alternative measure for quantitative SPECT, capturing the status of metabolically active features while mitigating image noise [14, 15]. Given that it can mitigate for differences in image quality and contrast recovery between imaging systems it is also well-suited to multi-centre studies [16]. All three SUV metrics are used in clinical practice depending on the objective of the measurement, but it is important to understand the advantages and the limitations of each SUV metric.

There are many image-related factors, both controllable and uncontrollable, that must be considered when performing optimisation of quantitative SPECT-CT. Most of these factors will lead to negative bias (an underestimation of the actual activity concentration) in the quantitative values while others will lead to positive bias (an overestimation of activity concentration). Assuming that reconstruction corrections, such as measured attenuation and scatter, and a robust cross-calibration between the radionuclide calibrator and gamma camera are performed correctly, negative bias is largely due to partial volume effects from limitations in spatial resolution. Positive bias is mainly attributed to Poisson noise, arising from relatively low pixel values due to the constraints of scan time and patient administered activity, but may also be associated with reconstruction artefacts in some situations. Some technical factors that will influence these negative and positive bias factors are given in Table 1.

**Table 1:** Controllable and uncontrollable factors that influence the quantitative accuracy of activity concentration measurements obtained from SPECT-CT images

Factor	Controllable?	Impact of quantitative accuracy
Administered activity	Sometimes	For SUV_max_ and other regions based upon threshold of SPECT voxel values, positive bias can occur due to changes in noise level Note that geometric regions, manually delineated on whole organs or lesions where all encompassed SPECT voxel values are averaged, are less susceptible to variations in image noise levels It should be noted that for diagnostic applications, the administered activity is defined locally while, for therapeutic applications, it is likely to be either a fixed activity or defined by the planned therapy absorbed dose for the patient
Acquisition time	Yes	Positive bias from image noise—see comments above regarding administered activity
Collimator	Yes	Negative bias due to degrading spatial resolution
Matrix size	Yes	Negative bias due to changes in spatial sampling Positive bias due to changes in noise
SPECT orbit radius	Yes	Negative bias due to degrading spatial resolution for larger radii
Number of updates (product of iterations and subsets) for iterative reconstruction	Yes	Negative bias due to under-converged image Positive bias due to image noise
Post-reconstruction smoothing filter	Yes	Negative bias due to additional image blurring Positive bias due to control of image noise
Dead-time	No	Negative bias due to dead-time effects at very high count-rate levels
Lesion/organ size and shape	No	Negative bias due to degrading spatial resolution for small volumes
Organ-to-background contrast	No	Variable due to changes in spill-over from surrounding activity
Organ location	No	Negative bias due to increasing distance from the detector
Patient movement	No	Negative bias due to increasing image blurring

The optimisation process of quantitative SPECT-CT must evaluate the influence of the controllable variables on the accuracy and reproducibility of image-derived measurements. Furthermore, the parameters should be chosen such that they minimise, as far as possible, the influence of the non-controllable factors. It is essential that this is characterised using suitable phantom data, which will allow errors in the measurement to be determined.

This section describes the considerations needed from both the image acquisition and reconstruction processes in achieving reliable quantitative data from SPECT-CT images. It should be highlighted that centres intending to perform quantitative SPECT-CT should have suitable equipment to assess system performance with a minimum of uniform phantom to assess calibration and an IEC image quality (IQ) Phantom, a Jaszczak Phantom or similar to characterise activity concentration recovery coefficients (ACR) for various object sizes.

### Scanner calibration and characterisation

The differentiating factor between traditional SPECT-CT and quantitative SPECT-CT is the sensitivity calibration factor. Hence, the first step is to determine a reliable cross-calibration factor between a radionuclide calibrator and the gamma camera. The exact technique may differ depending on the system manufacturer and the radionuclide being used.

A prerequisite for scanner calibration is a robust measurement of activity in a radionuclide calibrator. Activities must be traceable to national and international standards. International guidelines recommend accuracies of 5–10% for diagnostic, and 5% for therapeutic radionuclides [17, 18]. The use of activity measurements traceable to primary standards is not common practice in all countries [19], even though larger variabilities have been observed in international comparison exercises [20]. Therefore, radionuclide calibrators should be regularly maintained and calibrated against a primary standard for the radionuclide and measurement geometry of interest following available good practice guidelines [21, 22].

There are two distinct considerations that will influence the accuracy of quantitative data extracted from reconstructed SPECT-CT images. These are the activity calibration and characterisation of the system. It is important to appreciate the difference in these concepts. Calibration of a system is the act of including the activity calibration factor of the system to produce activity concentration measurements in Bq/mL. Characterisation will describe the performance of a given measurement technique, for example SUV_max_, derived from images obtained from a specific combination of acquisition and reconstruction parameters. Both will be impacted by the acquisition and reconstruction parameters chosen as has been described in Table 1.

Clearly, a key component of quantitative SPECT-CT is the accuracy of the calibration factor relating reconstructed counts from the scanner to activity measured in a radionuclide calibrator whose measurements should be traceable to a primary standard. Errors in this calibration factor will lead to systematic bias in quantitative values derived from the images. It is important to appreciate that this is a two-stage process: one of calibration, and another of verification of quantitative accuracy following the calibration process. The calibration process itself will vary according to the manufacturer’s requirements, which may range from a petri-dish source planar measurement; a long-lived sealed point source planar measurement; or volumetric measurements from a large uniformly filled phantom acquired, reconstructed and corrected using the same parameters used for patient imaging. Regardless of the method, it is recommended to perform this calibration using the method and with the frequency suggested by your system manufacturer. If no recommendations are given, the calibration should be performed at least annually, or after any major changes to hardware or software using a volumetric approach given that it better represents imaging conditions [23, 24]

Once calibration has been performed, it must be verified before clinical use. This should be performed using a large uniform volumetric phantom filled with the appropriate radionuclide and acquired on the SPECT-CT with a clinical imaging protocol. If the initial calibration is performed using a volumetric method, the verification scan should not be performed immediately after the calibration using the same phantom as any errors in the activity measurement or phantom filling will simply transfer across to the verification scan. The verification should be performed with a freshly filled phantom to allow a test of the entire process of activity measurement, phantom filling and image acquisition. The importance of establishing a good technique of measuring activity in the radionuclide calibrator should not be neglected and should mirror the measurement approach used for patients. If patient injections are performed in specific syringe sizes and diluted to a given volume, this should be replicated when measuring the activity for the calibration and verification phantom scans. The outcome of verification is to ensure that the measured activity concentration agrees with the true activity concentration.

**Fig. 1:**
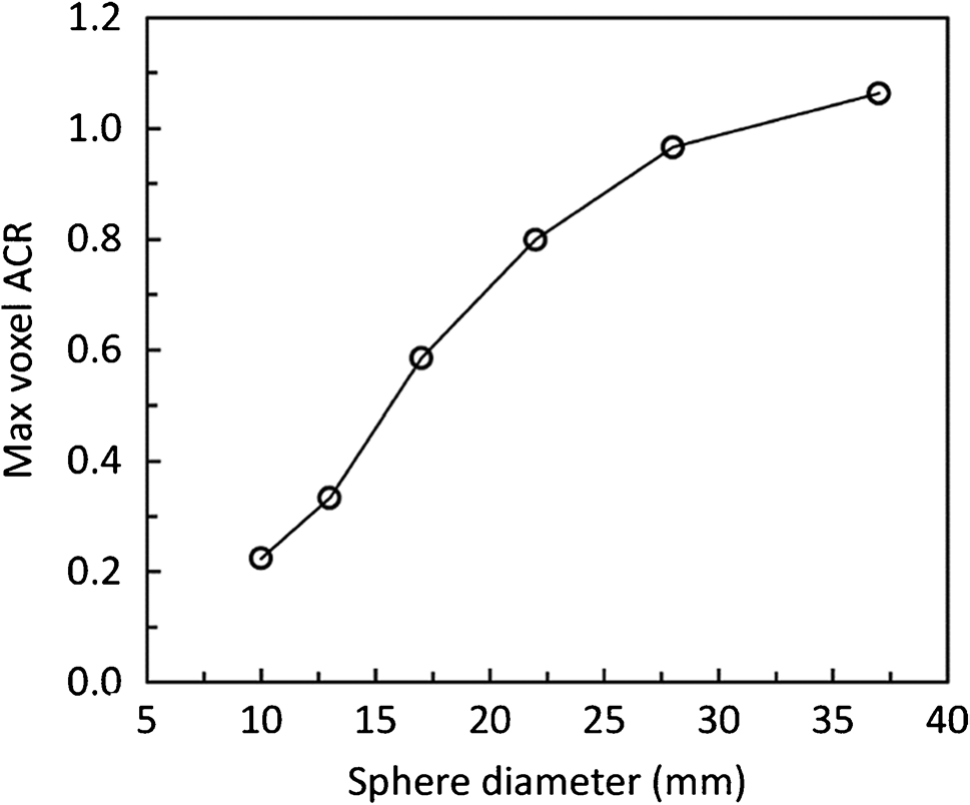
Example of an activity concentration recovery (ACR) curve obtained from the six hot spheres of a NEMA IEC image quality phantom filled with 10:1 contrast to background. In this example, activity concentration was measured by the maximum voxel value, which is akin to SUV_max_

Once a system has been calibrated, it is essential to characterise the relationship between object size and measurement accuracy. This relationship is commonly demonstrated in the form of activity concentration recovery (ACR) curves, which are commonly derived in PET-CT [25]. These measurements are performed using phantoms, such as the NEMA IEC IQ or Jaszczak phantoms, with a representative range of fillable volumes. Recovery curves are specific to the contrast and measurement of activity concentration that has been performed e.g., SUV_max_, SUV_mean_ and hence care should be taken to ensure clinically relevant measurements are taken when characterising the system. Figure 1 shows an example of an ACR curve.

### Acquisition

Acquisition parameters can vary depending on the patient investigation being performed and, on the characteristics of the camera. Each centre should therefore optimise its own acquisition parameters. Some steps that should be followed in the acquisition process are:

- Steps should be taken to limit the possibility of patient motion. It is important that the patient remains in the same position during both the CT and SPECT acquisition to ensure good image registration and accurate CT attenuation correction.
- The optimal collimator will depend on the radionuclide being imaged. Relevant imaging guidelines should be followed when choosing an appropriate collimator.
- Step and shoot or continuous acquisition mode of acquisition can be used. The latter can offer a 1–2 min saving on scanning time over 60 rotation angles.
- Detector auto-contouring is advised to minimise the distance between the detectors and patient to provide optimal spatial resolution. However, for some applications detectors can be kept at a fixed but close distance.
- Acquisition should typically be performed with opposing detectors at 180° from one another, but for cardiac applications a 90° configuration may be used.
- A pixel size smaller than half the full width at half maximum (FWHM) spatial resolution of the system for the radionuclide used is recommended to ensure appropriate spatial sampling. Commonly, a matrix size of 128 × 128 is used. It should be noted that decreasing the pixel size results in a noisier image.
- The number of projections is recommended to be similar to the matrix size (e.g. 120–128 projections for a 128 × 128 matrix) to ensure appropriate angular sampling. Cardiac applications using a 90° detector configuration may use a reduced arc and number of projections, although distortion and inaccurate quantification will occur when there are insufficient projection data—typically distant from the heart.
- The time per projection will depend on the amount of radioactivity in the patient. As noise in the projection data follows a Poisson distribution, and in reconstructed data is much worse [26], imaging time must be high enough to reduce image noise as much as possible. If multiple fields of view (FOVs) are acquired, the time per projection may have to be decreased for patient comfort.

### Reconstruction

**Fig. 2:**
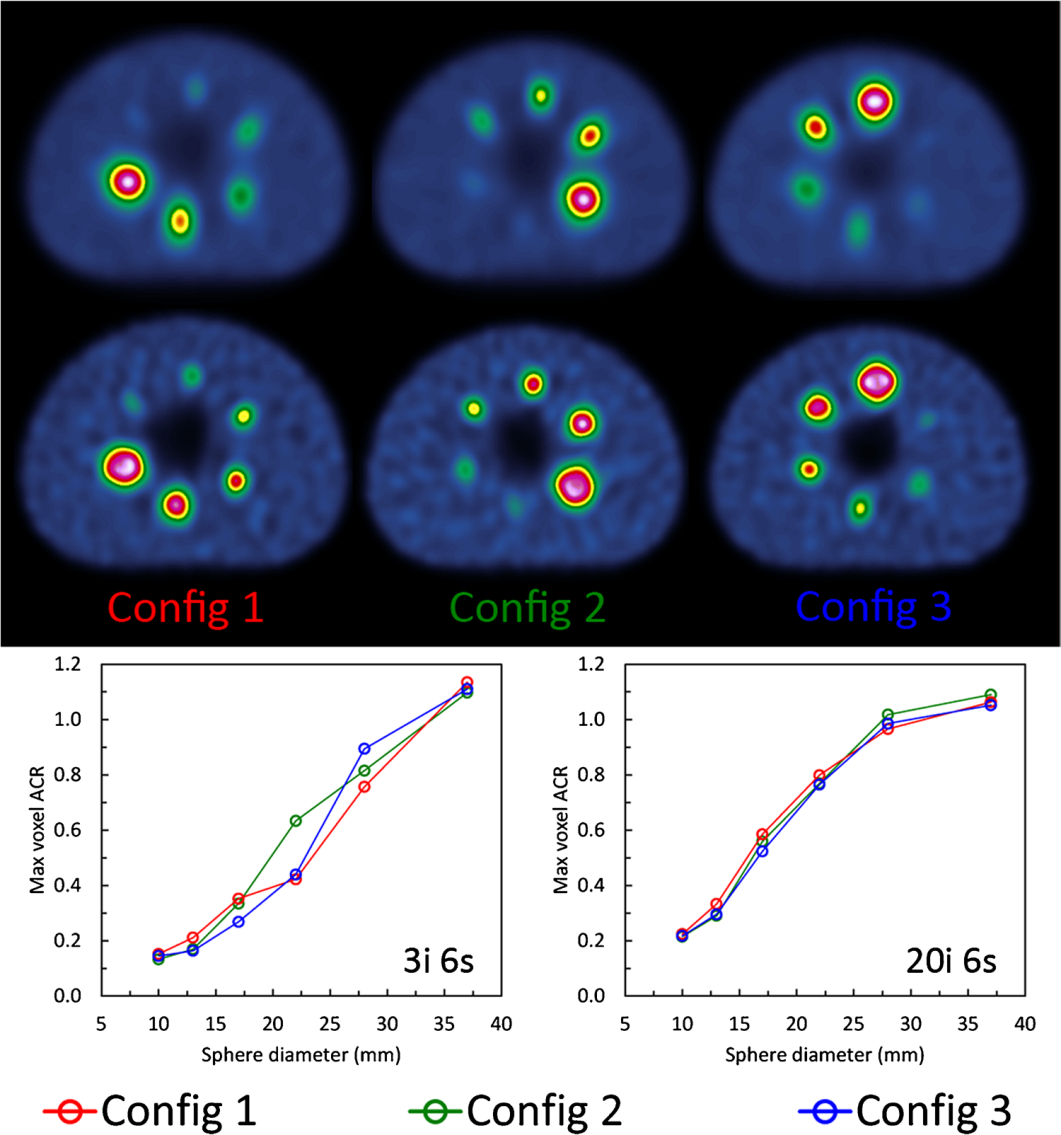
NEMA IEC image quality phantom filled with ^99m^Tc at 10:1 contrast in three different sphere configurations reconstructed with 3 iterations and 6 subsets (upper set of images) and 20 iterations and 6 subsets (lower set of images). The corresponding recovery curves are given below for maximum voxel ACR. Note that all phantom images shown have corrections for attenuation, scatter and collimator response included and a 10 mm Gaussian filter applied

Iterative methods are recommended to reconstruct the acquired SPECT projections. Normally, the algorithm used will be that included in the software provided by the vendor of the gamma camera; however, third-party algorithms are also available. For quantitative purposes, the number of updates, defined by the product of the number of iterations and subsets, may be greater than for reconstructions with qualitative purposes [27]. Preferably, that number should be obtained from phantom measurements in which the convergence of activity concentration is studied, paying attention not to reach excessive noise levels or introduce image artefacts, such as Gibbs artefacts from the use of resolution modelling. Ideally, such optimisation should be performed using anthropomorphic phantoms which mimic the clinical situation, but such phantoms are not always available. If NEMA IEC IQ or Jaszczak phantoms are used as part of the optimisation process, it should be noted that SPECT recovery curves are far more dependent on sphere position than PET. This is likely due to the variable detector radius acquisition resulting from auto-contouring [28] and so it is recommended that multiple sphere configurations are evaluated. Figure 2 shows an example of how the variability of ACR can be aligned across different sphere configurations when sufficient updates are performed.

### Corrections

#### Attenuation correction

Attenuation correction based on CT data should be used for quantitative SPECT-CT. The CT images should be converted to a map of attenuation coefficients for the appropriate radionuclide energy and incorporated into the iterative reconstruction process [29]. It must also be possible to measure the full path length of the gamma-ray from the point of emission to the patient boundary on the attenuation map. If the attenuation map is truncated, either by way of large patient habitus or inadequate CT field of view, then the attenuation correction will not be accurate and quantitative errors will occur. Hence, care should be taken when positioning the patient to minimise the likelihood of truncation. Where appropriate, and when available, it is recommended to use metal artefact correction algorithms [30] to produce more suitable attenuation maps [31].

#### Scatter correction

To correct for scattered gamma-rays present within the photopeak window, multiple energy window scatter correction methods are typically applied, although model-based scatter correction can also be used if available [32]. Dual-energy window methods can be applied if there are no emissions above the photopeak of the radionuclide used; otherwise, a triple-energy window method should be implemented. Smoothing of the scatter window image may also be beneficial to reduce propagation of image noise from the correction to the reconstructed image. It is important to validate scatter correction techniques using appropriate phantoms containing areas of no activity, surrounded by uniform activity, to demonstrate that the algorithms do not over-correct the final images.

#### Resolution modelling

Resolution modelling is available in most modern reconstruction software and partially compensates for the limited spatial resolution due to the collimator and the detector by incorporating a depth-dependent collimator response model in the projection operation of the iterative reconstruction method [33]. It is recommended that resolution modelling techniques are used if available to improve quantification.

#### Decay correction

Understanding how and when decay correction is applied is important in quantitative SPECT-CT. Given the relatively long physical half-life of most SPECT radionuclides, its application to ensure differences are accounted for in the acquisition of the first and last projection are relatively minor. One exception being ^81m^Kr SPECT-CT where differences in acquired projection counts will be large and application of such corrections essential. In multiple SPECT field of view studies where the study may take up to 1 h, decay correction should also be performed to ensure consistency of relative pixel values across all acquired projections.

A second area where decay correction is important is in the measurement of SUV metrics. With SUV normalising uptake to injected activity, it is important that SPECT data acquired several hours or even days later accounts for the decay (especially for radionuclides such as ^177^Lu). Ensuring clocks on scanners and in injection rooms are consistent is an important element of ensuring this is done appropriately.

#### Partial volume (sphere and non-sphere)

Resolution modelling will rarely fully compensate for the limited spatial resolution of the gamma camera. Hence, to quantify the activity in the volumes of interest (VOI), post-reconstruction corrections can be applied. These recovery coefficients are commonly obtained from phantoms with spherical inserts of known volume that are filled with known activity concentrations [23]. Note that the applicability of this method has limitations for non-spherical volumes. For some anatomical regions (e.g. brain), methods based on anatomical information such as the geometric-transfer matrix method can be applied [34]. While partial volume corrections are not generally available with commercial scanner software and therefore not clinically available, ‘homemade’ site applied corrections can be useful for dosimetry, and some research applications. Note that for clinical use, any site written software should be in alignment with appropriate medical device legislation [35].

#### Dead-time

For acquisitions performed soon after administering therapeutic amounts of radiopharmaceuticals, e.g. treatment of neuroblastoma with ^131^I-mIBG or ^177^Lutetium peptide therapy of neuroendocrine tumours, dead-time correction may need to be applied [36, 37]. The non-linearity of the count rate will not be the same for every projection angle. Therefore, the dead-time correction should preferably be applied on each projection acquired [38]. However, for simplicity, an average correction could be applied based on the count rate averaged over all projections.

### Reconstruction post-filtering

**Fig. 3:**
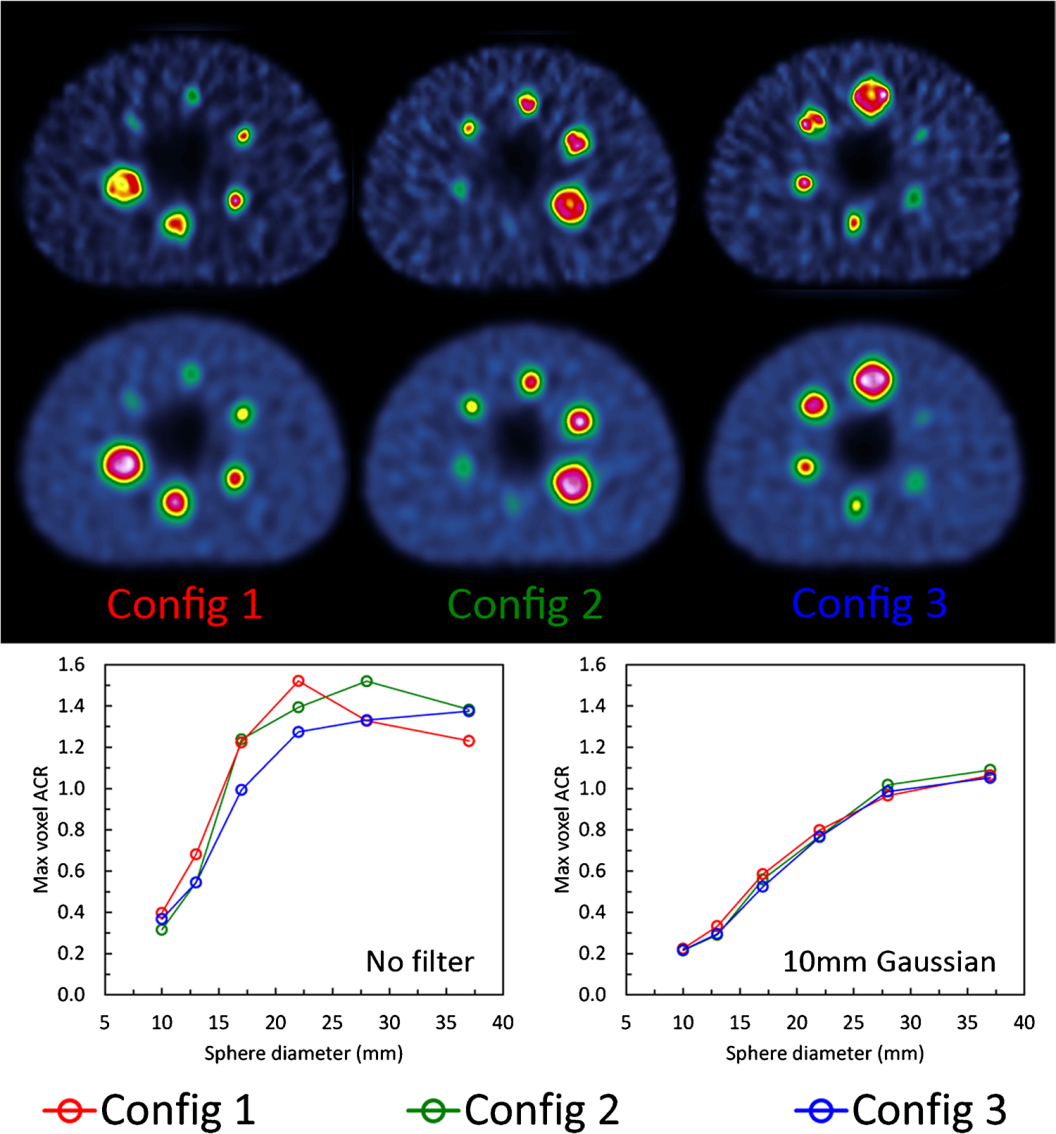
NEMA IEC image quality phantom filled with ^99m^Tc at 10:1 contrast in three different sphere configurations reconstructed with 120 updates with no post-filter (upper set of images) and a 10 mm Gaussian post-filter (lower set of images). The corresponding recovery curves are given below for maximum voxel ACR

The application of a post-reconstruction smoothing filter will inevitably degrade the spatial resolution and exaggerate partial volume effects. It is therefore often assumed that filtering should not be applied to images for quantitative applications. However, the application of a post-filter is intended to control the degree of noise in the image and hence the potential positive bias that may arise from noise. The application and choice of a post-reconstruction filter is dependent on the desired outcome from the image and nature of the measurement. In situations where large regions are drawn, such as organ delineation, and where mean activity concentration measurements are derived from all voxels, then a post-filter is unlikely to be beneficial. However, in situations where images are drawn on small objects and maximum voxel values are extracted, i.e. where noise is a more significant influence, then a post-filter can be helpful in ensuring a greater level of consistency. Figure 3 demonstrates how the application of a 10 mm Gaussian post-filter can substantially improve the consistency of measurements of activity concentration. The filter has been applied to images reconstructed with the same number of iterative updates. It is shown that the post-filter increases the degree of negative bias in the smaller objects, but removes the positive bias, due to noise, present in the larger spheres of the unfiltered images.

For clinical reporting, many nuclear medicine images are produced according to a visual preference of the clinician, and hence it may be appropriate to create a second reconstruction that is optimised for quantification.

## Quality control and harmonisation

### Quality control requirements

Acceptance testing and quality control of SPECT-CT systems for SPECT and CT components should follow international guidelines [21, 39]. Given the importance of attenuation correction for quantitative accuracy, focus should also be given to the alignment between SPECT and CT, which must be checked periodically. The sensitivity calibration workflow can differ significantly between vendors. It is therefore recommended that the local physics team should determine the appropriate frequency in which to perform verification scans of sensitivity, based on manufacturer recommendations. The sensitivity calibration must be repeated with major changes in software or hardware.

### The need and methods of harmonisation

Accurate and reproducible measurements of radioactivity are essential for quantitative SPECT-CT imaging, enabling the comparison of results from multi-centre studies [40]. This can be achieved by establishing traceable results [41] that can be related to a reference through a documented unbroken chain of calibrations, each contributing to the measurement uncertainty [42]. However, the uncertainties at all stages of the imaging acquisition and processing chain are not presently known or measured, partly due to the variations in available correction methods, reconstruction algorithms and the black-box nature of the available software. Full traceability is therefore not presently achievable, often stopping with the activity measurements in the radionuclide calibrator prior to use in phantom or patient studies.

Harmonisation of ^18^F-FDG PET-CT has been addressed by the EANM Research Ltd EARL accreditation programme [43], and a similar approach is currently in development for SPECT-CT. The initiative in SPECT-CT was started following the recent interest in absolute quantification for this modality, mainly driven by the need of dosimetry following radionuclide therapy. Many investigators have explored the variability of activity quantification in interlaboratory and multi-centre studies for ^99m^Tc [44–47], ^123^I [48, 49], ^131^I [49, 50], ^133^Ba [51], ^177^Lu [24, 52, 53], ^223^Ra [54], highlighting the need for harmonisation protocols.

Accounting for differences in availability of local resources, a set of minimum requirements to harmonise SPECT-CT imaging across centres is recommended:

- A radionuclide calibrator traceable to a national standards laboratory.
- A suitable and accessible phantom set to calibrate the scanner and to assess partial volume effects through ACR curves following the recommendations described earlier.
- Standard operating procedures (SOP) for traceable phantom preparation, image acquisition and reconstruction, assessment of partial volume effects, outlining volumes of interest and reporting of results [19, 55].
- A verification phantom study is recommended to assess the quantitative accuracy across centres. This can be a more realistic geometry, e.g. a circular or elliptical cylindrical phantom to match the clinical condition under consideration [24, 49].

All the scans must be acquired and reconstructed with the same protocols used for the specific clinical condition. For dosimetry following radionuclide therapy and to enable a quantitative comparison between centres, it is recommended to calculate the uncertainties in the recovery coefficients following EANM guidelines [56].

## The path to clinical use

Quantitative SPECT-CT is an emerging imaging technology, but, as with any new technology, its success depends on whether routine clinical applications can be identified. Not every technical evolution is automatically translated into wide clinical acceptance, and depends on issues including impact, ease-of-use, cost, availability and an adequately trained workforce [57]. If quantitative SPECT-CT is here to stay, it should answer clinically relevant questions and impact patient treatment and outcome.

Recent developments in nuclear medicine in association to new theranostic approaches [58] support the use of SPECT-CT for some radionuclides, such as ^177^Lu and ^131^I to visualise the efficacy of treatments. Quantitative SPECT-CT can quantify how much activity is delivered to each tumour lesion and organ at risk, and consequently, the absorbed dose. This may be of high clinical importance for personalised medicine, even though confirmation in future studies is needed.

The estimation of absolute activity concentration may also be attractive for several other purposes: (1) to deliver a reliable diagnosis, (2) for accurate therapy response monitoring, (3) for prognosis and to guide patient management decisions, (4) to improve the reproducibility of interpretations, (5) to allow comparison of data between centres (6) and to facilitate (semi)automatic analysis. Initial applications reported in the literature include the assessment of skeletal conditions (e.g. bone metabolism, detection of bone metastases, mandibular condyle asymmetry), coronary artery disease, amyloidosis and parkinsonism.

For quantitative SPECT-CT, it will therefore be essential to define the limitations of the measures produced by this technique. As different applications have different accuracy/precision requirements, understanding the technology’s limitations will help guide the focus towards areas with the highest likelihood of successful clinical implementation. Optimisation for a range of applications, radionuclides, geometries and activities will also be necessary, as will the transferability of results. The cost-effectiveness of the technique with regards to the humanistic and societal outcomes must also still be proven, which is beyond the scope of these guidelines. However, these guidelines should aid in the design of clinical trials with the appropriate methodology required to demonstrate the value of the technique.

## Clinical use cases

### Dosimetry

Until recently, radionuclide therapies were dominated by the use of radioactive iodine, which has been used successfully for therapy of benign and malignant thyroid diseases for over 80 years. However, even though it is not necessarily recommended in guidelines [59, 60], due to the ease of application, in most countries thyroid patients are prescribed fixed activities selected by the clinical team based on the underlying diagnosis, pathology and staging. The only application of SPECT-CT so far in this setting is the post-therapy scan, which is mainly assessed using visual interpretation. ^131^I dosimetry has shown some success as an indication of potential response [61], and for treatment planning [62] with further success evident using ^124^I, albeit with PET [63].

In recent years, there has been an increasing interest in new radionuclide therapy concepts. This was initially driven by the development and approval of ^177^Lu-Dotatate (Lutathera®), triggered by the compelling data of the NETTER-1 study [64]. The use of ^177^Lu PSMA in the treatment of metastatic prostate cancer has also shown great promise as demonstrated in the recently published VISION study [65], with other novel radiopharmaceuticals also in development [58]. While rather slowly growing neuroendocrine tumours have a large therapeutic index which may not require dosimetry, the development of theranostic concepts in more aggressive tumours underlines the need for dosimetry during therapeutic development and potentially at clinical application. Furthermore, whereas initial clinical trials employ fixed activities, increasing response rates by escalating the applied activities based on imaging should be considered. For those patients not yet responding to radionuclide therapy, the individualisation of the therapeutic activity using pre- and post-therapeutic dosimetry may be of benefit. Special attention must be given to the robustness, reproducibility and simplicity of these techniques. Once this prerequisite is provided, prospective clinical trials can be conducted to potentially prove the superiority of individually calculated tumour absorbed dose derived activities versus standard activities. The potential clinical relevance of personalised therapeutic activities can be seen in the phase II ^177^Lu-PSMA data by Hofman et al. [66]. Despite carefully selecting patients using PSMA- and ^18^F-FDG-PET 43% of patients did not show a prostate-specific antigen (PSA) decrease of > 50%. One possibility is to salvage some of these patients by applying higher activities that are more focused on tumour absorbed dose. There is also evidence that personalised treatment may work using ^177^Lu-DOTATATE. In a recent study (ILUMINET), it was found that individualising the therapeutic approach can increase the response rate of the therapy without causing significant toxicity [67]. Another important application of quantitative SPECT-CT is the prospective development of new theranostic agents that might be applied with a smaller therapeutic window. Quantitative SPECT-CT will play a key role in the optimisation between acceptable toxicity to normal organs and maximal absorbed dose to the tumour.

### Cardiology

#### Perfusion

**Fig. 4:**
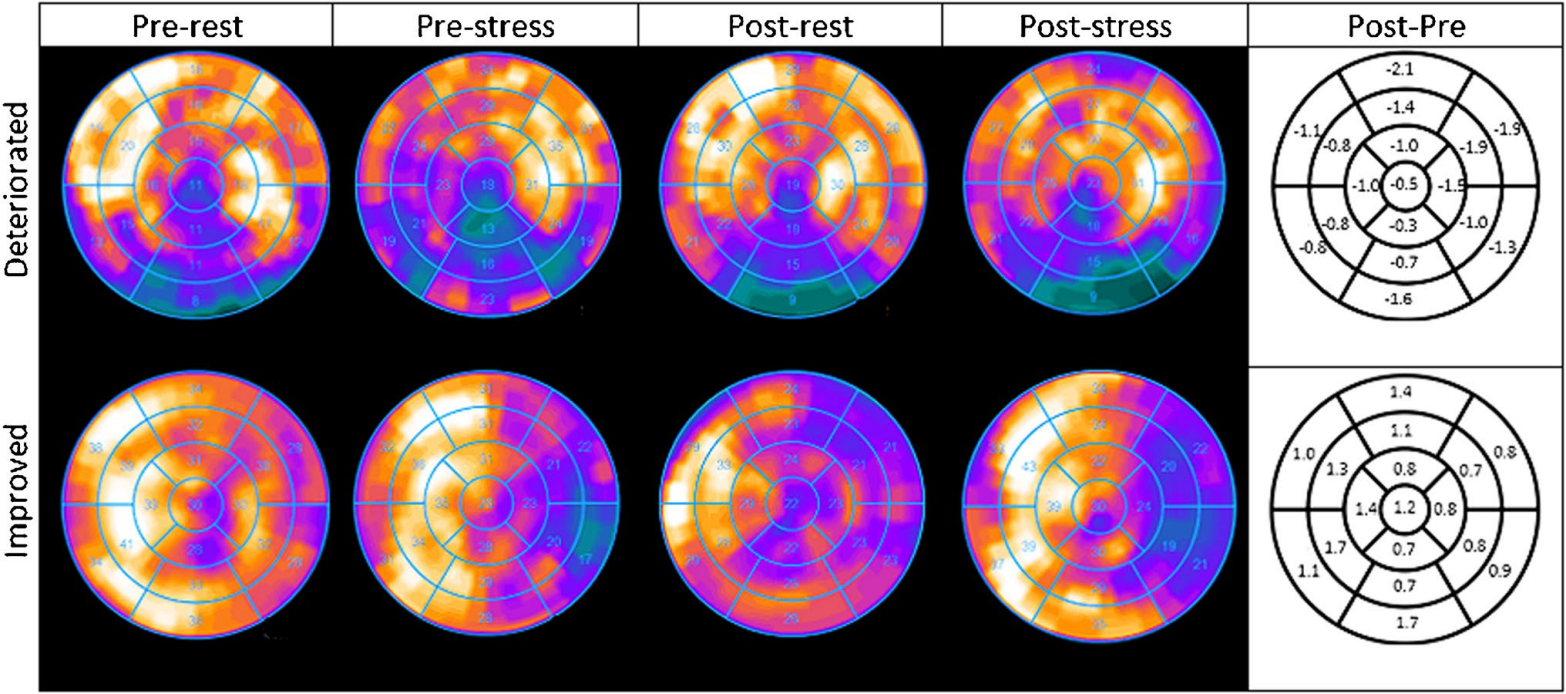
Bull’s eye plots of two patients in kBq/mL. The rest and stress plots are depicted pre- (left columns) and post-treatment (right columns). Moreover, the Bull’s eye plot on the right displays the difference in uptake from the subtracted (stress-rest) scans post minus pre-treatment. The patient in the top row was considered clinically deteriorated and the patient in the bottom row was reported as improved. Since the distribution of perfusion abnormalities of the patient in the top row in particular does not vary a lot, visual comparison is difficult without quantification

Myocardial perfusion imaging using either ^99m^Tc-tetrofosmin or ^99m^Tc-sestamibi SPECT-CT is the most extensively validated imaging modality to evaluate the presence and severity of coronary artery disease, and is routinely used to manage treatment strategies [68]. The interpretation of myocardial perfusion scintigraphy (MPS) is most often based on relative myocardial perfusion and may underestimate the severity of ischemia in case of global hypoperfusion. To overcome this limitation of MPS, quantitative SPECT-CT could be helpful to provide absolute instead of relative measurements of radiotracer myocardial uptake [12, 69, 70]. Two illustrative patient cases of response monitoring using ^99m^Tc-tetrofosmin SPECT-CT in patients with coronary artery diseases are demonstrated in Fig. 4.

#### Amyloidosis

Amyloidosis is a multisystem disease that is characterised by extracellular deposition of abnormally folded protein fibrils, resulting in progressive organ dysfunction, commonly affecting the heart [71]. It includes three pathophysiologic amyloid types: primary light chain (AL) and transthyretin-related amyloidosis associated with (variant ATTR) or without a TTR gene mutation (wild-type ATTR). In case of a clinical suspicion of cardiac amyloidosis, based on clinical symptoms, specific demographics and serum biomarkers, scintigraphy with bone-seeking radiotracers such as ^99m^Tc-3,3-diphosphono-1,2-propanodicarboxylic acid [DPD], ^99m^Tc-pyrophosphate [PYP] or ^99m^Tc-hydroxymethylene diphosphonate [HMDP] is highly sensitive and specific in the early identification of ATTR cardiac amyloidosis when a plasma cell dyscrasia is excluded [72]. Also in asymptomatic TTR gene carriers at initial evaluation, in the screening for cardiac amyloidosis in case of new symptomatic heart failure, and in the follow-up of TTR gene carriers or patients with known ATTR amyloidosis and new or worsening cardiac symptoms, a cardiac phosphate scan is a key diagnostic technique [73]. Using this procedure, it is possible to offer a non-invasive diagnosis, reducing the need for endomyocardial biopsy [74]. Several visual and relative scoring systems have been used to quantify amyloid burden [71, 75]; however, there is a need for a more accurate measurement technique using quantitative SPECT-CT to diagnose and characterise cardiac amyloidosis at the earliest opportunity, to be able to accurately monitor response to therapy and to predict patient prognosis [71, 76]. This is very important, especially in the case of ATTR amyloidosis, which is a progressive and fatal cardiomyopathy for which several promising therapies are in development.

### Neurology

#### DaTScan

To differentiate essential tremor from Parkinson’s syndrome dopamine transporter (DaT), ^123^I FP-CIT SPECT can be helpful. However, Parkinson’s syndrome can have several causes, such as Parkinson’s disease, drug-induced parkinsonism, vascular parkinsonism, multiple system atrophy, progressive supranuclear palsy, corticobasal degeneration and Lewy body dementia. The clinical presentation of Lewy body dementia is very similar to that of Parkinson’s disease and of Alzheimer’s disease which complicates the clinical diagnostic process. Particularly during the early stages of disease, the clinical diagnosis lacks accuracy. Accurate discrimination of the different diagnoses is key, since patient management, treatment and the course of these disease entities substantially differ. Presynaptic dopaminergic imaging helps clarify the differential diagnosis between neurodegenerative parkinsonian syndromes and non-dopamine deficiency etiologies of parkinsonism [77]. DaT SPECT scans are commonly interpreted visually, by using scoring systems, or by means of calculating the specific binding ratio, which is an index to measure DAT density. It is, however, challenging to discriminate age-related physiological reduction from pathological reduction in DAT availability. Poorly reproducible interpretations and measurements are occasionally experienced in clinical practice [78] and data from different centres need to be interpreted with caution, recognising that the specific binding ratio represents an ‘index’ rather than a ‘true’ value [79]. Absolute quantification of radiopharmaceutical uptake may be an alternative measure to improve diagnostic accuracy [13].

### Bone imaging

Technetium-99m labelled bisphosphonates accumulate in newly formed bone and enable visualisation of bone turnover. Many conditions are associated with pathological bone turnover, and bone SPECT-CT using these tracers is an established and powerful diagnostic tool in their diagnosis and management [80].

#### Bone metastases

According to a recent review, applications in bone disease resides as the leading clinical application of quantitative SPECT-CT [81]. One of the earliest studies identified an average SUV of approximately 6 for the spine for MDP [82]. Building on this experience, an SUV threshold between 9 and 10 was proposed in a multicentre study to differentiate benign from malignant bone lesions, akin to the Hounsfield thresholds proposed on CT imaging to distinguish bone metastases from enostosis [83, 84]. A validated set of normal ranges can improve the reader’s diagnostic certainty and facilitate automated delineation algorithms to measure disease burden. In the setting of treatment response, data suggest that SUV analysis yields more consistent results than visual assessment [85]. Nevertheless, early experience from the field of PET has shown that absolute thresholds are only reliable when harmonised acquisitions and reconstructions are used, and confirmatory prospective multicentre studies are needed.

#### Mandibular condyle asymmetry

**Fig. 5:**
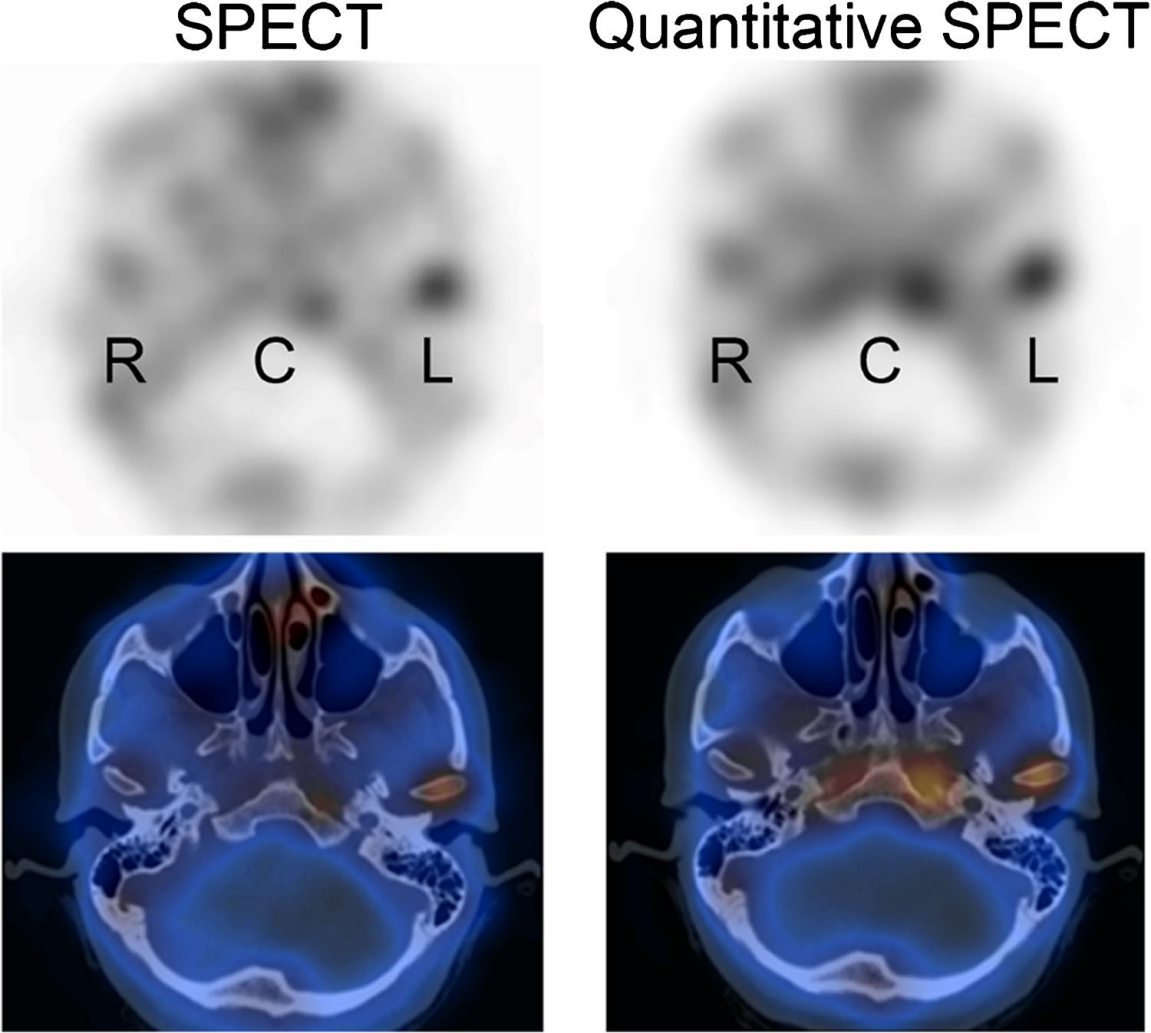
Bone SPECT-CT in a patient with mandibular growth asymmetry to the right side, showing unilateral increased uptake in the left mandibular condyle (L). The right (R) condyle and clivus (C) are shown as reference regions. In the left image SPECT-only reconstruction of counts without correction (L/Total: 60.0%; L/Clivus: 55.4%), while on the right quantitative SPECT-CT images (kBq/mL) with attenuation, scatter and resolution modelling (L/Total: 52.6%; L/Clivus: 45.5%). No partial volume correction was used. Using the traditional threshold of > 55% between the affected side and the total activity in the condyles or clivus would yield different interpretations in this example (most likely by the correction of activity in the clivus due to attenuation correction). This illustrates the need of redefining diagnostic cut-offs with novel reconstruction methods, ideally moving towards age-standardised absolute thresholds of normal condylar activity

Assessing the mandibular condyle’s growth activity in the presence of facial asymmetry is crucial to determine the optimal timing for surgery or the corrective surgical technique required. For many years, bone scintigraphy (with planar or SPECT acquisitions, and more recently also SPECT-CT) has been used with relative quantification methods comparing uptake in the affected side with the contralateral condyle alongside a reference region in the skull base or spine. These ratios are a more reliable predictor of residual growth activity than volumetric assessment using CT [86]. Nevertheless, discordant results with SPECT have been published, suggesting that some sources of error may not be adequately addressed using relative uptake assessment [87]. Absolute quantification of tracer uptake on SPECT-CT may potentially resolve these uncertainties. A clinical example is given in Fig. 5.

#### Osteoarthritis and arthroplasty

While arthroplasty is a highly effective treatment for osteoarthritis, the condition itself needs improved biomarkers to select patients for treatment, and joint replacement surgery is associated with a low complication rate but with a potentially high patient impact. The use of SUVs on bone SPECT-CT has been suggested as a promising tool to assess the severity of knee osteoarthritis, and preliminary data show that quantitative reporting of abnormalities seen in hip and knee prostheses is superior to qualitative assessment [88, 89]. Further experience is needed to assess the true potential of absolute tracer quantification in this setting.

## Future perspectives

Quantitative SPECT-CT is here now—primarily driven by the development of theranostics, but also for bone and other diagnostic uses. It is clear that there is a huge drive in radionuclide therapy from academia and industry, with many new agents in development or on the way to market [58]. This will be the driver for future quantitative SPECT-CT. Also in diagnostics, the nuclear medicine physician/radiologist who has become accustomed to quantitative measures in PET imaging will rightly demand the same benefits over visual interpretation in SPECT. The key area holding this back currently is the overtly long scanning times, and for some indications, the reporting time overhead of 3D versus planar imaging too. But there are developments here as well. Ring detector systems are already on the market which can collect SPECT projection data more rapidly [90], while image processing [91] and traditional and artificial intelligence (AI) based reconstruction algorithms can better handle noisy data that come from shorter duration SPECT acquisitions [92–94]. As for the reporting burden, we are already seeing AI algorithms supporting image interpretation in PET, which will no doubt be translated to SPECT imaging in due time [95, 96]. Quantitative SPECT-CT has a bright future. With these guidelines we set a framework for protocols and clinical use cases that will help take it forward.

## Declarations

### Liability statement

This guideline summarizes the views of the EANM Physics and Dosimetry Committees. It reflects recommendations for which the EANM cannot be held responsible. The recommendations should be taken into context of good practice of nuclear medicine and do not substitute for national and international legal or regulatory provisions
